# Bioactive metabolites of botanical drugs in the treatment of intervertebral disc degeneration-a review of promising therapeutic candidate

**DOI:** 10.3389/fphar.2025.1700077

**Published:** 2026-01-12

**Authors:** Le Qi, Xinming Fan, Jiabing Sun

**Affiliations:** Department of Orthopedics, The First Affiliated Hospital of Harbin Medical University, Harbin, China

**Keywords:** bioactive metabolites of botanical drugs, intervertebral disc degeneration, preclinical research, promising therapeutic candidate, traditional Chinese medicine

## Abstract

**Background:**

Intervertebral disc degeneration (IVDD) refers to the structural degeneration of intervertebral discs that occurs with aging or overuse, including annulus fibrosus rupture, nucleus pulposus dehydration, reduced proteoglycan content, and decreased elasticity. The bioactive metabolites of botanical drugs (BMBDs) refer to the chemical substances derived from plants that can exert specific physiological effects on living organisms, including the human body. Various types of the BMBDs regulate key protein targets and signaling pathways, demonstrating effects such as alleviating nucleus pulposus cell inflammation and oxidative stress levels, inhibiting extracellular matrix degradation, and regulating nucleus pulposus cell autophagy and apoptosis.

**Methods:**

All experimental information and summaries used in this review were acquired from peer-reviewed articles in the relevant fields. The PubMed, Web of Science (WOS), Google Scholar, and China National Knowledge Infrastructure (CNKI) databases were searched for relevant articles. Information on the manual classification and selection of BMBDs that protect against IVDD is included in this review.

**Results:**

The literature review identified multiple studies on the characteristics of BMBDs, which delay IVDD from various aspects through a wide range of key targets and signaling pathways.

**Conclusion:**

This review summarizes the pharmacological effects and mechanisms of different types of BMBDs in the treatment of IVDD, providing a theoretical foundation for further pharmacological research and the development of new drugs for treating IVDD, as well as strong theoretical support for future clinical applications.

## 1 Introduction

Intervertebral disc degeneration (IVDD) is a degenerative condition of the intervertebral disc primarily driven by aging, chronic mechanical stress, trauma, and other factors. It results in the dehydration of the nucleus pulposus (NP), leading to a loss of the disc’s elasticity and tensile strength, which diminishes its ability to support significant physical activity. This degeneration can cause spinal stenosis, instability, and symptoms such as low back and leg pain, often leading to disability globally (Zàaba et al., 2025). With the global aging population and lifestyle changes—including sedentary behavior and excessive physical exertion—the incidence of IVDD is rising, creating substantial economic burdens on individuals and society. This condition predominantly affects individuals over the age of 60 (Wu et al., 2025). Low back pain (LBP), one of the most common manifestations of disc degeneration, affects an estimated 619 million people worldwide as of 2020, with projections indicating 843 million cases by 2050 (Dou et al., 2025; Wang et al., 2025).

**FIGURE 1:**
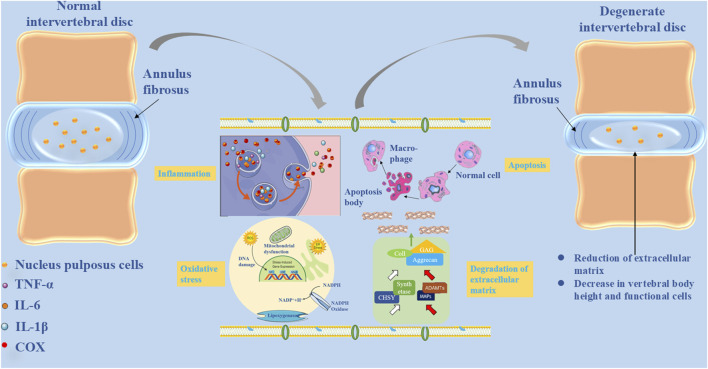
The pathophysiological basis of intervertebral disc degeneration, including nucleus pulposus cell inflammation, oxidative stress, apoptosis, and extracellular matrix degradation.

Several risk factors contribute to IVDD, including aging, mechanical injury, obesity, height, axial spinal overload, and smoking. LBP related to lumbar disc degeneration is primarily caused by disc degeneration, compression, and herniation (Mai et al., 2025). The pathophysiological mechanisms underlying IVDD are as follows: 1) Cellular and environmental changes make the spine more susceptible to mechanical stress and gene expression dysregulation, which can alter the structure and function of lumbar intervertebral discs, compromising spinal stability and increasing vulnerability to external forces (Tsuchiya et al., 2025). 2) IVDD represents an aberrant cell-mediated response to structural disruptions between the annulus fibrosus (AF) and NP, progressively worsening over time. Degenerative processes involve changes in cell composition and structure within the NP, contributing to disc degeneration (Chen et al., 2025). 3) Elevated catabolic activity in the extracellular matrix (ECM) of the intervertebral disc leads to reduced hydration and anabolic activity within the NP, further driving disc degeneration (Zhan et al., 2025). 4) Dysregulation of ECM homeostasis triggers inflammation, with inflammatory cytokines and neurogenic mediators such as neurotrophic factors promoting hyperinnervation of sensory nerve fibers within the disc. This process induces nociception and contributes to the development of disc-derived LBP (Yao et al., 2025), as illustrated in Figure 1.

Current treatment options for IVDD are categorized into conservative and interventional approaches. Conservative treatments include bed rest, non-steroidal anti-inflammatory drugs, muscle relaxants, hot compresses, and physical therapy. While these measures provide short-term relief from LBP, they do not address the underlying progression of disc degeneration (Mohd Isa et al., 2022; Yang et al., 2024). Interventional treatments encompass epidural steroid injections, nerve root blocks, and surgical options such as discectomy, interbody fusion, and disc replacement. When conservative treatments fail, surgery is typically considered the primary intervention. However, surgical procedures carry certain disadvantages, including damage to the original disc structure, partial loss of mechanical properties, recurrence, and adjacent segment degeneration (Kim et al., 2022). Both conservative and surgical treatments offer limited and often unsustainable benefits, as they primarily focus on alleviating symptoms rather than halting or reversing the degenerative process. In recent years, novel treatment strategies have emerged, aimed at preventing IVDD and promoting regeneration. These include growth factor therapy, cell therapy, and gene therapy (Sono et al., 2024). Various studies have investigated the injection of transforming growth factors, bone morphogenic proteins (BMPs), insulin-like growth factors, nucleus pulposus cells (NPCs), bone marrow mesenchymal stem cells, and viral vectors (lentivirus, adenovirus, or adeno-associated virus) carrying connective tissue growth factor (CTGF), transforming growth factor-β3 (TGF-β3), and tissue inhibitor of metal protease 1 (TIMP-1) into degenerated intervertebral discs. These approaches aim to repair and regenerate the disc, but they are hindered by high costs and poor targeting (Lou et al., 2025). Thus, current treatments for disc degeneration remain limited and largely symptomatic, with ongoing research focused on the discovery of more effective and targeted therapies.

The bioactive metabolites of botanical drugs (BMBDs) refer to the collective term for chemical substances derived from plants and capable of exerting definite physiological effects on living organisms. They are the products of secondary metabolism in plants and constitute the modern scientific core of the therapeutic effects of botanical drugs. Traditional Chinese medicine (TCM) metabolites contain multiple BMBDs, the efficacy of which is the result of the synergistic action of multiple BMBDs contained within them. A large number of studies have shown that the comprehensive therapeutic effects of BMBDs and the TCM metabolite prescriptions composed of them have a significant role in treating various diseases. For instance, ginsenosides, derived from ginseng, have been found to address spinal cord injury (SCI) through multiple mechanisms: anti-inflammation, anti-apoptosis, antioxidative stress, and inhibition of glial scar formation. These metabolites also alleviate osteoporosis by affecting osteoclast and osteoblast activity (Qi et al., 2022; Liu et al., 2024). TCM, including metabolites formulations, extracts, and active monomers, can treat cardiovascular diseases (CVD) through various pharmacological mechanisms, such as inducing mitophagy, making them promising candidates for the development of cardiovascular drugs with fewer side effects and improved efficacy (Wang et al., 2024). In the treatment of chronic atrophic gastritis, precancerous lesions, and gastric cancer, TCM metabolites are beneficial for long-term use, as they cause no significant side effects, improve overall physical health, and enhance immune function (Liu X. et al., 2025).

This review explores the potential of BMBDs and in alleviating IVDD through its diverse mechanisms, providing a comprehensive theoretical foundation for its clinical application.

## 2 Specific varieties of BMBDs

The BMBDs form the material basis for the therapeutic effects of TCM metabolites, and the latter are composed of these metabolites in an orderly and complex application system according to the theories. Therefore, we will separately summarize the multi-target, multi-pathway and multi-effect therapeutic roles of both in the treatment of IVDD.

### 2.1 Active single BMBDs

#### 2.1.1 Ginsenosides

Ginsenosides, a group of key natural triterpene saponins, are identified as the primary active metabolites responsible for the pharmacological effects of ginseng. Nearly 200 distinct ginsenosides have been isolated from ginseng plants and heat-processed ginseng products (Qi et al., 2022). These metabolites are generally categorized into two subtypes: protopanaxadiol (PPD) and protopanaxatriol (PPT). Examples of PPD-type ginsenosides include Rb1, Rb2, Rb3, Rc, Rd, Rh2, Rg3, and F2, while PPT-type ginsenosides include Re, Rf, Rg1, Rg2, and Rh1. These metabolites have shown potential in the treatment of a wide range of diseases, including diabetes, cancer, stress, inflammation, immune modulation, and cardiovascular disorders. In 2019, Zhang et al. (2019) demonstrated for the first time that ginsenoside Rd could inhibit IL-1β-induced inflammation and degradation of intervertebral disc chondrocytes by enhancing the ubiquitination of the IL-1 receptor accessory protein (IL1RAP). Bioinformatics analysis suggested that the proteins neural precursor cell expressed, developmentally downregulated protein 4 (NEDD4), Casitas B-cell lymphoma (CBL) and itchy E3 ubiquitin protein ligase Gene (ITCH) are likely to target IL1RAP. Among ginsenosides, Rg1 has been the most extensively studied in relation to IVDD. Research indicates that Rg1 inhibits apoptosis of NPCs, through the Wingless-Type MMTV Integration Site Family (Wnt)/β-catenin, Yes-associated protein 1 (YAP1)/Transcriptional coactivator with PDZ-binding motif (TAZ), and NF-κB (NF-κB) signaling pathways. Additionally, Rg1 promotes ECM synthesis, inhibits ECM degradation, and reduces inflammation in the NP tissue, thereby playing a pivotal role in alleviating disc degeneration (Yu et al., 2020; Yang et al., 2022; Yu et al., 2024). Beyond Rg1, NR1 has also been shown to enhance cellular function and reduce pyroptosis in NPCs through the NF-κB/Nucleotide-binding oligomerization domain, leucine-rich repeat and pyrin domain-containing 3 (NLRP3) pathway, while also alleviating mechanical and thermal hyperalgesia in IVDD mouse models (Tang et al., 2021).

**FIGURE 2:**
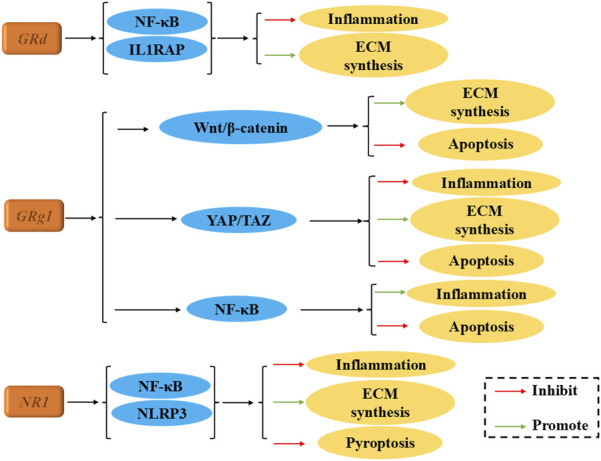
The therapeutic effects and underlying mechanism of Ginsenosides on IVDD.

In summary, ginsenosides exhibit significant therapeutic potential in the management of IVDD, as illustrated in Figure 2; Table 1. With the integration of precision drug delivery systems and biomaterial carriers, ginsenosides may represent a promising new approach to the treatment of IVDD in the future.

**TABLE 1:** Detailed information on ginsenoside for the treatment of IVDD.

Drug name	Effective dose	Route of administration	Vitro duration	Animal	Animal model	Mechanism	Signal path or receptor	References
Rg1	• Vitro: 20, 50, 100 μg/mL • Vivo: 10, 20, 40 mg/kg/d	• Intraperitoneal injection	• 8 weeks	• 60 rats	• Remove the paraspinal musculature and the supraspinous and interspinous ligaments from C2-C7	• Promote ECM synthesis • Inhibit apoptosis of nucleus pulposus cells (NPCs)	• Wnt/β-catenin	Yu et al. (2020)
Rg1	• Vitro: 25, 50, 100 μM • Vivo: 20, 40, 80 mg/kg/d	• Intragastrically administrated	• 4 weeks	• 50 female Sprague-Dawley (SD) rats	• AF puncture surgery in Co7/8	• Inhibit the secretion level of inflammatory factors and redox activity • Inhibit degradation of ECM • inhibit apoptosis of NPCs	• YAP1/TAZ	Yang et al. (2022)
Rg1	• Vitro: 20, 50, 100 μM • Vivo: 20, 40, 80 mg/kg/d	• Intraperitoneal injection	• 4 weeks	• 50 male SD rats	• Remove the paraspinal musculature and the supraspinous and interspinous ligaments from C2-C7	• Inhibit apoptosis of NPCs • Inhibit the secretion level of inflammatory factors	• NF-κB	Yu et al. (2024)
Notoginsenoside R1 (NR1)	• Vitro: 10, 25, 50 μM • Vivo: 20 mg/kg/d	• Intraperitoneal injection	• -	• 30 male SD rats	• AF puncture surgery	• Promoted the release of ECM • Decrease the expressions of proinflammation cytokines • Suppress cell pyroptosis	• NF-κB/NLRP3	Tang et al. (2021)

#### 2.1.2 Quercetin

Quercetin, a natural flavonoid present in a variety of plants, is part of the polyphenol family and is known for its significant biological activities, including antioxidant, anti-inflammatory, and anti-tumor effects. It is commonly found in apples, onions, grapes, tea, broccoli, and other fruits and vegetables, and is a key metabolite in many dietary supplements (Carrillo-Martinez et al., 2024). The molecular structure of quercetin enables it to effectively scavenge free radicals, inhibit oxidative stress, and modulate multiple signaling pathways to exert anti-inflammatory effects. Moreover, research has shown that quercetin can inhibit cancer cell proliferation, induce apoptosis, and offer cardiovascular protection, antiviral properties, and immunomodulatory functions (Kamal et al., 2024). In recent years, quercetin’s potential applications in metabolic diseases, neurodegenerative disorders, and cancer prevention and treatment have attracted significant attention.

Apoptosis of NPCs and the dysregulation of ECM homeostasis are key contributors to the pathogenesis of IVDD. In 2020, Wang et al. first reported that quercetin has a significant impact on alleviating IVDD. They demonstrated that quercetin prevented NPC apoptosis and ECM degeneration by promoting SIRT1-dependent autophagy. Further experiments showed that the autophagy inhibitor 3-methyladenine (3-MA) reversed this protective effect, suggesting quercetin as a novel and effective treatment for IVDD (Wang et al., 2020). Subsequent studies have expanded on these findings, indicating that quercetin can suppress inflammation, apoptosis, oxidative stress, and ECM degradation in NPCs (Shao et al., 2021; Zhang et al., 2021; Yu H. et al., 2025). Additionally, Wu et al. (Wu et al., 2025) reported in 2025 that quercetin inhibits pyroptosis in NP cells through Tripartite Motif Containing 31 (TRIM31), a key protein, further elucidating its mechanism in alleviating IVDD. In cell-based studies, Ren and Zhao et al. shifted focus to nucleus pulposus mesenchymal stem cells (NPMSCs) (Zhao et al., 2023; Ren et al., 2024). Their results demonstrated that quercetin significantly reduced oxidative stress, apoptosis, and senescence in NPMSCs by modulating key signaling pathways, including hypoxia inducible factor 1A (HIF1A) and miR-34a-5p/silent information regulator 1 (SIRT1). These findings were further corroborated in an IVDD rat model.

**FIGURE 3:**
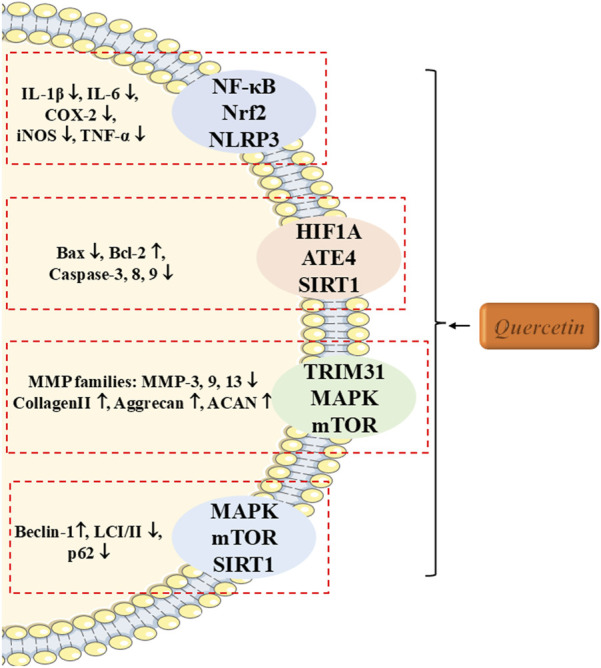
The therapeutic effects and underlying mechanism of Quercetin on IVDD. ATE, Arginyltransferase 1.

Overall, quercetin, as a natural and low-toxicity metabolite, holds substantial promise for the treatment of IVDD, as illustrated in Figure 3; Table 2. Future research should focus on exploring its long-term effects in humans and optimizing its administration to improve bioavailability, positioning quercetin as a potential new therapeutic strategy to delay or reverse IVDD.

**TABLE 2:** Detailed information on quercetin for the treatment of IVDD.

Drug name	Effective dose	Route of administration	Vitro duration	Animal	Animal model	Mechanism	Signal path or receptor	References
Quercitin	• Vitro: 10, 20, 40 μM • Vivo: 15, 30 mg/kg/2w	• Intraperitoneal injection	• 8 weeks	• 24 C57BL/6 mice	• Remove the spinous processes in L2-L6	• Alleviate inflammation • Maintain homeostasis of ECM • Inhibit pyroptosis	• TRIM31/NLRP3	Wu et al. (2025)
Quercitin	• Vitro: 100, 200, 300 mM	• -	• -	• -	• -	• Decreased endoplasmic reticulum (ER) stress-related apoptosis	• PERK-eIF2α-ATF4	(Yu Z. et al., 2025)
Quercetin	• Vitro: 5, 10, 20 μM • Vivo: 20 mg/kg/2w	• Intervertebral disc injection	• 2 weeks	• 24 C57BL/6 mice	• AF puncture surgery in Co8/9	• Alleviate oxidative stress, apoptosis, and loss of viability in nucleus pulposus mesenchymal stem cells (NPMSCs)	• HIF1A	Ren et al. (2024)
Quercetin	• Vitro: 100 μM • Vivo: 100 mg/kg/d	• Intragastrically administrate	• 4 weeks	• 15 male SD rats	• AF puncture surgery in Co6/7	• Alleviate senescence changes of (NPMSCs)	• miR-34a-5p/SIRT1	Zhao et al. (2023)
Quercetin	• Vitro: 10, 20 μM • Vivo: 100 mg/kg/d	• Intragastrically administrate	• 4 weeks	• 24 male SD rats	• AF puncture surgery in Co7/8	• Inhibit senescence associated secreted phenotype (SASP) factors expression and senescence phenotype in NPCs • Ameliorate the progression of IDD	• Nrf2/NF-κB	Shao et al. (2021)
Quercetin	• Vitro: 15, 25 μM • Vivo: 100 mg/kg/d	• Intragastrically administrate	• 4 and 8 weeks	• 36 male SD rats	• AF puncture surgery	• Alleviate oxidative intracellular ROS levels • Alleviate apoptosis • Promote ECM stability • Activate autophagy	• p38 MAPK/mTOR	Zhang et al. (2021)
Quercetin	• Vitro: 100 μM • Vivo: 100 mg/kg/d	• Intragastrically administrate	• 8 weeks	• 18 male SD rats	• AF puncture surgery in Co7/8	• Inhibit the apoptosis of NPCs and ECM degeneration • Promote autophagy	• SIRT1	Wang et al. (2020)

#### 2.1.3 Baicalein

Baicalin, a flavonoid extracted from the dried root of Scutellaria baicalensis Georgi, exhibits a broad spectrum of pharmacological activities, including anti-inflammatory, antioxidant, antibacterial, antiviral, hepatoprotective, anti-tumor, and neuroprotective effects (Wen et al., 2023). Recently, it has garnered attention for its potential in alleviating IVDD, with its mechanisms primarily involving anti-inflammatory and antioxidant effects, as well as the regulation of ECM metabolism.

Baicalin effectively inhibits the release of pro-inflammatory cytokines such as tumor necrosis factor-α(TNF-α), interleukin-1 beta (IL-1β) and IL-6, thereby reducing the inflammatory response in the intervertebral disc microenvironment and delaying the degenerative process (Wu et al., 2025). Additionally, baicalin protects NPCs from oxidative stress-induced damage by scavenging reactive oxygen species (ROS) and preserving cell viability and function (Liu W. et al., 2023). In terms of matrix metabolism, baicalin inhibits the expression of matrix-degrading enzymes like matrix metallopeptidase 13 (MMP-13) and A Disintegrin and Metalloproteinase with Thrombospondin motifs-5 (ADAMTS-5), while promoting the synthesis of collagen II (Col2α1) and aggrecan, thereby maintaining the structural integrity of the intervertebral disc (Gan et al., 2025). Animal studies further confirmed that baicalin treatment significantly improved intervertebral disc height and signal intensity, while reducing histopathological degeneration scores. Baicalin may also exert protective effects through the regulation of autophagy and apoptosis-related pathways, such as the Mitogen-activated protein kinases (MAPK) signaling pathway (Jin et al., 2019).

Although the clinical application of baicalin is hindered by its low water solubility and bioavailability, advancements in drug delivery systems, such as hydrogels or nanoformulations, hold promise for overcoming these limitations. In conclusion, baicalin, as a natural multi-target drug, has significant potential in the prevention and treatment of IVDD.

#### 2.1.4 Berberine

Berberine, an isoquinoline alkaloid derived from Coptis coptidis and Phelloberia amurensis, is characterized by its bright yellow crystals and bitter taste. This potent natural metabolite has been shown to possess a wide array of pharmacological effects, with its mechanism of action involving multi-target regulation (Li et al., 2023).

In IVDD, berberine effectively inhibits NPCs apoptosis through various signaling pathways, including AMPK/mammalian target of rapamycin (mTOR)/Unc-51 like autophagy activating kinase 1 (Ulk1), NF-κB, and Immunoglobulin-Regulated Enhancer 1 (IRE1)/c-Jun N-terminal kinase (JNK) (Lu et al., 2019; Luo et al., 2019; Huang et al., 2022). This suggests that its pharmacological mechanism forms a complex network. In addition to inhibiting apoptosis, berberine has been shown to prevent IL-1β-induced ECM degradation by regulating ECM-related enzymes and factors. It also significantly activates autophagy, which is otherwise inhibited by IL-1β (Huang et al., 2022).

**FIGURE 4:**
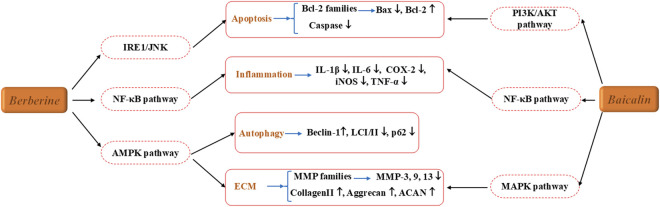
The therapeutic effects and underlying mechanism of Berberine and Baicalin on IVDD.

Due to its multi-target action profile and good safety profile, baicalein and berberine has become a focus of research in modern natural drug development, as illustrated in Figure 4; Table 3. It holds significant potential for the prevention and treatment of chronic metabolic and degenerative diseases.

**TABLE 3:** Detailed information on baicalein and berberine for the treatment of IVDD.

Drug name	Effective dose	Route of administration	Vitro duration	Animal	Animal model	Mechanism	Signal path or receptor	References
Baicalin	• Vitro: 0, 25, 50, 100, 200 μM • Viro: 100 mg/kg/d	• Intragastrically administrate	• 4 weeks	• 24 male C57BL/6 L mice	• AF puncture surgery in Co6/7, Co7/8, Co8/9	• Downregulate inflammatory factors and catabolic factors • Upregulate anabolic factors	• p38 MAPK	Wu et al. (2025)
Baicalein	• Vitro: 50, 100, 200 mg/mL • Vivo: 0.5%, 1%	• Silk fibroin solution	• 14 days	• -	• Skin incision	• inhibit the senescence of NPCs	• NF-κB	Gan et al. (2025)
Baicalein	• Vitro: 0, 5, 10, 20, 30, 40, 50, 60 μM	• -	• -	• -	• -	• Inhibit apoptotic signaling and catabolic activity in NPCs	• PI3K/AKT	(Liu Y. et al., 2023)
Baicalein	• Vitro: 5, 10, 20 μM	• -	• -	• -	• -	• Inhibit inflammation	• NF-κB and MAPK	Jin et al. (2019)
Berberine	• Vitro: 5,10,15,20, 25 μM	• -	• -	• -	• -	• Prevent apoptosis	• NF-κB	Lu et al. (2019)
Berberine	• Vitro: 0,1,2,4,8 μM • Vivo: 150 mg/kg/d	• Intraperitoneal injection	• 8 weeks	• 24 female SD rats	• AF puncture surgery in Co7/8	• Prevent apoptosis	• IRE1/JNK	Luo et al. (2019)

#### 2.1.5 Curcumin

Curcumin is a natural polyphenolic metabolite extracted from plants such as Zingiberaceae and Araceae, with its primary active metabolite found in Curcuma longa. Known for its excellent antioxidant and anti-inflammatory properties, curcumin has attracted considerable attention in the fields of medicine and healthcare (Kotha and Luthria, 2019). Curcumin can neutralize free radicals, reduce inflammatory reactions, and may play a role in preventing and improving cardiovascular and neurodegenerative diseases (Corrêa Carvalho et al., 2024).

**FIGURE 5:**
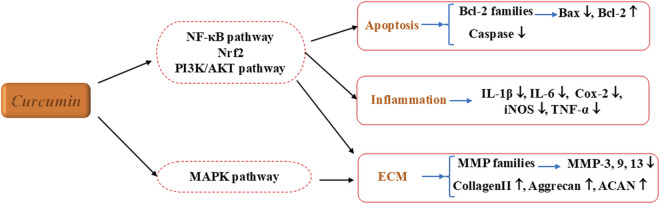
The therapeutic effects and underlying mechanism of Curcumin on IVDD. Nrf2, Nuclear factor erythroid 2-related factor 2.

IVDD, a leading cause of low back and leg pain, is associated with an inflammatory response. Curcumin has shown considerable pharmacological potential in treating IVDD. It can inhibit inflammatory signaling pathways, such as NF-κB, reducing the production and release of pro-inflammatory cytokines like TNF-α and IL-6. This decreases local inflammation in the intervertebral disc, alleviating pain and tissue damage (Hu et al., 2017; Cherif et al., 2019; He et al., 2021). Additionally, curcumin regulates cell metabolism by promoting the proliferation of NPCs, inhibiting apoptosis, and stabilizing cell numbers. It also affects ECM metabolism by reducing the degradation of collagen and proteoglycans by matrix metalloproteinases, while promoting their synthesis. These actions help maintain the structure and function of the intervertebral disc, making curcumin an effective agent in combating IVDD (Klawitter et al., 2012; Wan et al., 2025), as illustrated in Figure 5; Table 4.

**TABLE 4:** Detailed information on curcumin for the treatment of IVDD.

Drug name	Effective dose	Route of administration	Vitro duration	Animal	Animal model	Mechanism	Signal path or receptor	References
Curcumin	• Vitro: 10, 15, 20 μM	• -	• -	• -	• -	• Promote proliferation of chondrocytes • Inhibit apoptosis	• -	Wan et al. (2025)
Curcumin	• Vivo: 200 mg/kg/d	• Mix with food	• 5 weeks	• 30 male SD rats	• AF puncture surgery in L4/5	• Inhibit inflammation	• -	Hu et al. (2017)
Curcumin and o-Vanillin	• Vivo: 5 and 100 μM	• -	• -	• -	• -	• Increase matrix synthesis • Proliferate apoptotic cells	• Nrf2 and NF-kB	Cherif et al. (2019)
Curcumol	• Vitro: 0, 25, 50, 100, 200, 400 nM • Vivo: 10 mg/kg/d	• Intraperitoneal injection	• 4 weeks	• 80 male C57BL/6 L mice	• AF puncture surgery in Co6/7	• Alleviate the inflammation	• PI3K/Akt/NF-κB	He et al. (2021)
Curcuma	• Vitro: 25,50,100,250,500, 1,000 μg/mL	• -	• -	• -	• -	• Anti-inflammatory • Anti-catabolic effect	• JNK/p38 MAPK/and ERK	Klawitter et al. (2012)

To overcome challenges related to its bioavailability, researchers are exploring nanoformulations, structural modifications, and phospholipid complexes. As research progresses, the application of curcumin is expected to expand significantly.

#### 2.1.6 Other active single BMBDs

From both theoretical and practical perspectives, BMBDs provides distinct advantages for the individualized treatment of IVDD. In the realm of mechanistic research, cutting-edge technologies such as network pharmacology and metabolomics are enabling deeper analysis of BMBDs’ multi-metabolite, multi-target, and multi-pathway mechanisms. By identifying the core active substances in traditional formulations and elucidating specific molecular mechanisms—such as regulating the metabolism of intervertebral disc cells, inhibiting inflammatory responses, and promoting ECM repair—research can provide a robust theoretical foundation for the scientific application of BMBDs.

Astragaloside (Tian et al., 2022), Arctigenin (Ji et al., 2022), Andrographolide (Zhang et al., 2018), Palmatine (Yu H. et al., 2025) and Phillyrin (Chen et al., 2024) can significantly reduce the release of inflammatory factors, including IL-6, IL-1β, COX-2 and iNOX, by inhibiting the NF-κB signaling pathway. Icariin, Genkwanin and Wogonin can exert anti-inflammatory effects by regulating other signaling pathways, such as mitogen-activated protein kinase (MAPK) and toll-like receptor 4 (TLR4)/MyD88 (Deng et al., 2017; Fang et al., 2018; Hua et al., 2018; Hu et al., 2023; Li M. et al., 2024). In terms of anti-NPCs aging and apoptosis, Puerarin (Tang et al., 2023) can significantly reduce the apoptosis rate of NPCs and decrease the expression levels of apoptosis-related proteins. Sinomenine (Gao et al., 2019) and Naringenin (Li et al., 2016; Tang et al., 2025) can protect NPCs by reducing the secretion of aging-related phenotypes, including p16 and p21. These three pharmacological effects are achieved by activating the autophagy function of NPCs. It can be seen that autophagy plays an important role in this aspect. However, none of them have studied the relationship between mitochondrial autophagy with nucleus pulposus cells, which is also a future research direction. The studies of Wang, Li and Shao have shown that Polydatin, Tomatidine and Hyperforin can respectively alleviate oxidative stress by activating the Nrf2 pathway, inhibit ferroptosis by regulating the heme oxygenase-1 (HO-1)/glutathione peroxidase 4 (GPX4) pathway, and delay NPCs aging through a key target, transient receptor potential cation channel member 6 (TRPC6) channel (Wang et al., 2018; Li Z. et al., 2024; Shao et al., 2024). In addition, various BMBDs have proved to significantly inhibit ECM degradation and the activity of matrix metalloproteinases to promote the synthesis of collagen and proteoglycans (Li et al., 2015; Bai et al., 2022a, Bai et al., 2022b; Xu et al., 2022; Li M. et al., 2024).

**FIGURE 6:**
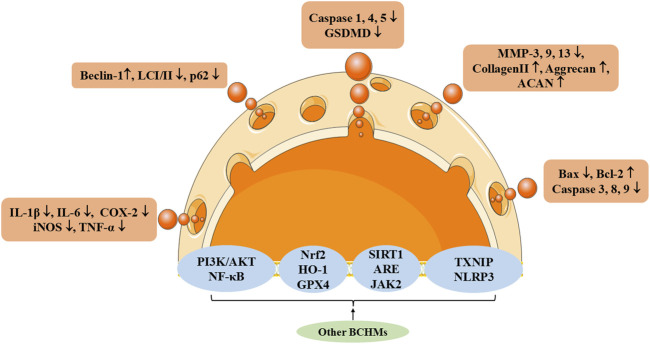
The therapeutic effects and underlying mechanism of other BMBDs on IVDD. TXNIP, Thioredoxin-Interacting Protein.

This part explores the specific mechanisms of other active single BMBDs in the treatment of IVDD, as illustrated in Figure 6; Table 5. As research into BMBDs continues to advance, it is expected to play an increasingly significant role in the prevention and treatment of IVDD, offering new hope for patients and contributing valuable insights to global medical practices.

**TABLE 5:** Detailed information on other active single BMBDs for the treatment of IVDD.

Drug name	Effective dose	Route of administration	Vitro duration	Animal	Animal model	Mechanism	Signal path or receptor	References
Astragaloside	• Vitro: 100 μM • Vivo: 50 mg/kg/d	• Intragastrically administrated	• 4 weeks	• SD rats	• AF puncture surgery in Co7/8	• Alleviate inflammation • Alleviate apoptosis • Alleviate ECM degeneration	• NF-κB	Tian et al. (2022)
Arctigenin	• Vitro: 10, 50 μM	• -	• -	• -	• -	• Inhibit apoptosis • Inhibit ECM degradation • Inhibit inflammation inNPCs	• miR-483-3p	Ji et al. (2022)
Andrographolide	• Vitro: 20 μM	• -	• -	• -	• -	• Inhibit apoptosis	• TLR4/MyD88/NF-κB	Zhang et al. (2018)
Cyanidin	• Vitro: 0, 2.5, 25, 50, 100, 200 μM	• -	• -	• -	• -	• Ameliorate apoptosis • Ameliorate ECM degradation	• Nrf2/HO-1	(Bai et al., 2022a)
Cyanidin	• Vitro: 25 μmol/L	• -	• -	• -	• -	• Attenuate degradation of ECM in NPCs	• Wnt/β-catenin	Xu et al. (2022)
Cyanidin	• Vitro: 25, 50 μM • Vivo: 50 mg/kg/d	• Intraperitoneal injection	• 8 weeks	• 24 male SD rats	• AF puncture surgery in Co4/5	• Attenuate the apoptosis of NPCs • Attenuate the degeneration of intervertebral disc	• JAK2/STAT3	(Bai et al., 2022b)
Emodin	• Vitro: 5, 10, 20 μM	• -	• -	• -	• -	• Mitigate the reduction of cell viability in NPCs • Reduce the production of ROS • Reduce apoptotic rate • Reduce inflammation	• NF-κB	Zhu et al. (2023)
Genkwanin	• Vitro: 40 μM • Vivo: 20 mg/kg/d	• Intraperitoneal injection	• 4 and 8 weeks	• 48 male SD rats	• AF puncture surgery in Co7/8	• Inhibit inflammation in NPCs • Promote extracellular matrix remodeling • Suppress cellular senescence and apoptosis	• ITGA2/PI3K/AKT • NF-κB • MAPK	(Li Z. et al., 2024)
Ginkgetin	• Vitro: 20, 40 μM • Vivo: 10 mg/kg/d	• Intraperitoneal injection	• 8 weeks	• 30 male SD rats	• AF puncture surgery in Co7/8	• Inhibit apoptosis, inflammation and disturbance of ECM	• NLRP3	Hu et al. (2023)
Hyperforin	• Vitro: 10, 30 μM • Vivo: 40 mg/kg/w	• Nucleus pulposus injection	• 4 weeks	• 18 male SD rats	• AF puncture surgery in Co5/7	• Rescue the inflammatory phenotype	• TRPC6	Shao et al. (2024)
Hyperoside	• Vitro: 10, 20, 50 μM	• -	• -	• -	• -	• Ameliorate inflammation • Ameliorate ECM degradation • Ameliorate apoptosis	• SIRT1/NF-κB • Nrf2/ARE	Xie et al. (2022)
Icariin	• Vitro; 0.1, 1, 10 µM	• -	• -	• -	• -	• Attenuate inflammatory response	• MAPK and NF-κB	Hua et al. (2018)
Icariin	• Vitro: 0.1, 0.5, 1, 5, 10, 20, 40, 50 μM	• -	• -	• -	• -	• Inhibit apoptosis	• PI3K/Akt	Deng et al. (2017)
Isofraxidin	• Vitro: 0, 10, 20, 40, 80 μM	• -	• -	• -	• -	• Suppress inflammation	• NF-κB	Su et al. (2019)
Kukoamine A	• Vitro: 10, 20, 40 μM	• -	• -	• -	• -	• Prevent loss of cell viability • Attenuate the apoptosis, ECM and inflammation	• P13K/Akt	Wang et al. (2022)
Ligustilide	• Vitro: 2, 5, 10, 20, 30 μM • Vivo: 10 mg/kg/d	• Intraperitoneal injection	• 8 weeks	• 36 SD rats	• AF puncture surgery	• Inhibit apoptosis • Suppress inflammatory mediators • Decrease inflammatory cytokines	• NF-κB	Wang et al. (2019)
Morin	• Vitro: 6.25,12.5,25,50,100, 200 nM • Vivo: 30 mg/kg/3d	• Intraperitoneal injection	• 12 weeks	• 12 male C57BL/6 L mice	• Remove the erector spinae muscle from L4/5	• Attenuate pyroptosis	• TXNIP/NLRP3	Zhou et al. (2021)
Naringenin	• Vitro: 0, 10, 20, 50, 100 μg/mL	• -	• -	• -	• -	• Attenuate senescence and degenerative phenotypes in NPCs	• IGFBP3	Tang et al. (2025)
Naringin	• Vitro: 20 μg/mL	• -	• -	• -	• -	• Promote the proliferation of degenerative human NP cells • Improve the recuperation of the cells from degeneration	• -	Li et al. (2016)
Palmatine	• Vitro: 20, 40, 80, 120, 160 μM • Vivo: 50, 100 mg/kg/d	• Intraperitoneal injection	• 4 weeks	• Male SD rats	• AF puncture surgery in Co7/8	• Inhibit degradation of ECM • Inhibit apoptosis of NPCs • Upregulate autophagy • Improve morphology and structure of AF and NP	• EB	(Yu Z. et al., 2025)
Phillyrin	• Vitro: 10, 20, 40, 80, 160, 320 μM • Vivo: 10 μM	• Intervertebral disc injection	• 4 weeks	• Male SD rats	• AF puncture surgery	• Inhibit the degeneration of ECM • Inhibit apoptosis • Inhibit the generation of reactive oxygen species (ROS)	• NF-κB	Chen et al. (2024)
Puerarin	• Vitro: 100, 200 mM • Vivo: 100, 200 mg/kg d	• Intraperitoneal injection	• 8 weeks	• 40 male SD rats	• AF puncture surgery in L4-6	• Decrease the apoptosis rate, levels of inflammatory factors	• TLR4/NF-κB	Tang et al. (2023)
Polydatin	• Vitro: 0, 200, 400 μM • Vivo: 50 mg/kg/d	• Intragastrically administrate	• 4 weeks	• 21 rats	• AF puncture surgery in Co7/8	• Suppress NPCs senescence • Promotes matrix homeostasis	• Nrf2	Wang et al. (2018)
Rhizoma drynariae total flavonoids	• Vivo: 62.5, 125, 250 mg/kg/d	• Intragastrically administrate	• 1 month	• 40 male SD rats	• Remove the paraspinal musculature and the supraspinous and interspinous ligaments from C2-C7	• Inhibit the inflammatory response and matrix degeneration	• MAPK	Zhao et al. (2021)
Shikonin	• Vitro: 4 μM	• -	• -	• -	• -	• Decrease apoptosis • Decrease inflammation	• NF-κB	Liu et al. (2021)
Sinomenine	• Vitro: 3.33 mM • Vivo: 25, 75 mg/kg/d	• Intraperitoneal injection	• 16 weeks	• 16 male SD rats	• AF puncture surgery in L5/6	• Ameliorate autophagy	• -	Gao et al. (2019)
Tanshinone IIA	• Vitro: 5 μM • Vivo: 30 mg/kg/12h	• Oral administration	• 3 weeks	• 60 female SD rats	• Remove the fascia and the multifidus muscle in L5/6	• Represses inflammatory response • Reduces radiculopathic pain	• IRAK-1 and NF-κB/p38/JNK	Li et al. (2015)
Tomatidine	• Vitro: 1,2, 4 μM • Vivo: 5, 10 mg/kg/w	• Intraperitoneal injection	• 8 weeks	• 24 male C57BL/6 mice	• Remove the supraspinous and interspinous ligaments in L3-L5)	• Promote ECM anabolism • Inhibit ECM catabolism • Reduce oxidative stress and ferroptosis	• Nrf2/HO-1/GPX4	(Li M. et al., 2024)
Wogonin	• Vitro: 100 μM • Vivo: 50 μM/2 μL	• Intervertebral disc injection	• 4 and 8 weeks	• 48 male SD rats	• AF puncture surgery in Co7-Co9	• Suppress inflammation	• Nrf2/ARE/MAPK	Fang et al. (2018)

### 2.2 TCM metabolites

#### 2.2.1 Bushen Huoxue Formula

Bushen Huoxue Formula is commonly used in clinical practice. Rooted in the theories of “kidney leading bone to pulp” and “activating blood circulation to remove blood stasis,” this formula has been shown through modern research to regulate bone metabolism, promote fracture healing, improve osteoporosis, and reduce IVDD. Additionally, it enhances immune function and improves microcirculation (Zhan et al., 2022). In clinical practice, physicians tailor the formula by making adjustments based on the patient’s specific disease and constitution, thus optimizing its therapeutic benefits.

The pharmacological effects of Bushen Huoxue Formula in the treatment of IVDD are multi-faceted. It regulates the metabolism of intervertebral disc cells, enhances the activity of NPCs, promotes cell proliferation, reduces apoptosis, and stabilizes the cell population within the intervertebral disc (Duan et al., 2023). Furthermore, it promotes the synthesis of proteoglycans, increases the content of key ECM, enhances the elasticity and compressive resistance of the intervertebral disc, and delays the degenerative process (Yang et al., 2019). In addition, Gao et al. (2022), Gao et al. (2024) reported that Bushen Huoxue Formula exhibits anti-inflammatory and antioxidant effects by inhibiting the production of inflammatory factors and ROS. It also restores mitochondrial function and autophagic flux (Gao et al., 2022; Gao et al., 2024).

As research continues, Bushen Huoxue Formula holds great promise for the treatment of IVDD and is expected to be further refined for precise clinical use as illustrated in Table 6. By optimizing the formula and clarifying dosage regimens, it offers hope for the rehabilitation of patients with IVDD.

**TABLE 6:** Detailed information on Bushen Huoxue Formula for the treatment of IVDD.

Name	Active metabolites	Effective dose	Route of administration	Vitro duration	Animal	Animal model	Mechanism	Signal path or receptor	References
Bushen Huoxue Formula	• Aconiti Lateralis Radix Praeparata (10 g) • Rehmanniae Radix Praeparata (20 g) • Radix Salviae (20 g) • Morindae Officinalis Radix (15 g) • Curculiginis Rhizome (10 g)	• Vitro: 15% • Vivo: 35, 70, 140 mg/kg/d	• Intragastrically administrate	• 4 weeks	• 30 SD rats	• AF puncture surgery in Co7/8	• Reduce the inflammatory levels • restore mitochondrial function by regulating the expression of antioxidant proteins • alleviate the apoptosis in NPCs	-	Gao et al. (2024)
BuShen HuoXue Formula	• Aconiti Lateralis Radix Praeparata (10 g) • Rehmanniae Radix Praeparata (20 g) • Radix Salviae (20 g) • Morindae Officinalis Radix (15 g) • Curculiginis Rhizome (10 g)	• Vitro: 20% • Vivo: 3 times/twice per day	• Intravenous infusion	• 1 h	• 30 Male SD rats	• Serum	• Alleviate the cycle blockage of NPCs after oxidative damage • encourage the growth and proliferation of NPCs • delay the aging of NPCs • improve the deteriorating microenvironment around NPCs • repaire oxidatively damaged NPCs	• TGF-β1/Smad	Duan et al. (2023)
Bushen Huoxue Formula	• Aconiti Lateralis Radix Praeparata (10 g) • Rehmanniae Radix Praeparata (20 g) • Radix Salviae (20 g) • Morindae Officinalis Radix (15 g) • Curculiginis Rhizome (10 g)	• Vitro: 15% • Vivo: 4 g/d	• Intragastrically administrate	• 6 weeks	• 30 SD rats	• Serum	• Restore mitochondrial function and autophagic flux • suppress excessive ROS production • protect ECM degradation and apoptosis	• AMPK/SIRT1	Gao et al. (2022)
Bushen Huoxue Formula	• Aconiti Lateralis Radix Praeparata (10 g) • Rehmanniae Radix Praeparata (20 g) • Radix Salviae (20 g) • Morindae Officinalis Radix (15 g) • Curculiginis Rhizome (10 g)	• Vitro: 10% • Vivo: 1.36 g/kg	• Oral administration	• 2 weeks	• SD rats	• serum	• Promote NPCs • Proliferate ECM synthesis	• Wnt	Yang et al. (2019)

#### 2.2.2 Duhuo Jisheng Decoction

Duhuo Jisheng Decoction is a well-established prescription commonly used to treat bi syndrome, liver and kidney deficiency, as well as deficiencies in qi and blood. Modern clinical research has demonstrated its significant therapeutic effects in the treatment of conditions such as rheumatoid arthritis, lumbar disc herniation, osteoarthritis, and other musculoskeletal diseases. Additionally, Duhuo Jisheng Decoction exhibits notable pharmacological activity in areas such as anti-inflammatory, analgesic, and immune regulation (Zhou et al., 2023). It remains many valuable metabolites and continues to play an important role in modern medical practice.

Duhuo Jisheng Decoction exerts its pharmacological effects through multiple targets and pathways in the treatment of IVDD. It effectively inhibits the release of inflammatory cytokines and the degradation of the ECM, IL-6 and TNF-α, thereby reducing local inflammation in the intervertebral disc (Liu et al., 2018; 2018). Furthermore, it regulates the metabolism of intervertebral disc cells through mechanisms such as autophagy and mitochondrial function. The decoction promotes the proliferation of NPCs, inhibits pyroptosis, and stabilizes the number of NPCs in the intervertebral disc. These effects are mediated through the Stromal Cell-derived Factor 1 (SDF1)/C-X-C chemokine receptor type 4 (CXCR4)-NFκB-NLRP3 and miR-494/SIRT3/mitophagy dual pathways (Guo et al., 2023; Liu W. et al., 2023). Additionally, Duhuo Jisheng Decoction can prevent ECM degradation and apoptosis by activating autophagy and inhibiting the P38/MAPK signaling pathway, which effectively delays degeneration in the intervertebral disc, as demonstrated in a puncture-induced IVDD rat model (Liu et al., 2020).

Duhuo Jisheng Decoction holds broad potential in the treatment of IVDD, as illustrated in Table 7. Its multi-metabolite and multi-target properties align well with the principles of modern precision medicine. Future research, including the application of advanced technologies such as metabolomics and network pharmacology, is expected to identify the core active substances of the formula and further elucidate its mechanism of action, enhancing its clinical application in treating IVDD.

**TABLE 7:** Detailed information on Duhuo Jisheng Decoction for the treatment of IVDD.

Name	Active metabolites	Effective dose	Route of administration	Vitro duration	Animal	Animal model	Mechanism	Signal path or receptor	References
Duhuo Jisheng Decoction	• Radix glycyrrhizae (6 g) • Panax ginseng (6 g) • Radix achyranthis bidentatae (6 g) • Eucommiae ulmoidis cortex (6 g) • Poria cocos (6 g) • Cortex cinnamomi (6 g) • Radix paeoniae alba (6 g) • Radix rehmanniae (6 g) • Radix angelicae sinensis (6 g) • Rhizoma chuanxiong (6 g) • Herba Asari (6 g) • Radix saposhnikoviae (6 g) • Radix gentianae macrophyllae (6 g) • Ramulus loranthi (6 g) • Radix angelicae pubescentis (9 g)	• Vivo: 0.32 g/100 g	• Intragastrically administrate	• 4 Weeks	• 30 male SD rats	• AF puncture surgery in Co6/7 and Co8/9	• Reduce inflammatory responses • suppress the expression of pyroptosis	SDF1/CXCR4/NF-κB/NLRP3	Guo et al. (2023)
Duhuo Jisheng Decoction	• Radix glycyrrhizae (6 g) • Panax ginseng (6 g) • Radix achyranthis bidentatae (6 g) • Eucommiae ulmoidis cortex (6 g) • Poria cocos (6 g) • Cortex cinnamomi (6 g) • Radix paeoniae alba (6 g) • Radix rehmanniae (6 g) • Radix angelicae sinensis (6 g) • Rhizoma chuanxiong (6 g) • Herba Asari (6 g) • Radix saposhnikoviae (6 g) • Radix gentianae macrophyllae (6 g) • Ramulus loranthi (6 g) • Radix angelicae pubescentis (9 g)	• Vitro: 300 μg/mL	• -	• -	• -	-	• Lessen apoptosis and mitochondrial dysfunction • activate mitophagy in NPCs	• miR-494/SIRT3/mitophagy	(Liu Y. et al., 2023)
Duhuo jisheng decoction	• Radix glycyrrhizae (6 g) • Panax ginseng (6 g) • Radix achyranthis bidentatae (6 g) • Eucommiae ulmoidis cortex (6 g) • Poria cocos (6 g) • Cortex cinnamomi (6 g) • Radix paeoniae alba (6 g) • Radix rehmanniae (6 g) • Radix angelicae sinensis (6 g) • Rhizoma chuanxiong (6 g) • Herba Asari (6 g) • Radix saposhnikoviae (6 g) • Radix gentianae macrophyllae (6 g) • Ramulus loranthi (6 g) • Radix angelicae pubescentis (9 g)	• Vitro: 200 μg/mL • Vivo: 10.8 g/kg/d	• Intragastrically administrate	• 8 weeks	• 45 SD rats	AF puncture surgery in Co6/9	• Reduce ECM degeneration • reduce apoptosis • activate autophagy	• p38/MAPK	Liu et al. (2020)
Duhuo Jisheng Decoction	• Radix glycyrrhizae (6 g) • Panax ginseng (6 g) • Radix achyranthis bidentatae (6 g) • Eucommiae ulmoidis cortex (6 g) • Poria cocos (6 g) • Cortex cinnamomi (6 g) • Radix paeoniae alba (6 g) • Radix rehmanniae (6 g) • Radix angelicae sinensis (6 g) • Rhizoma chuanxiong (6 g) • Herba Asari (6 g) • Radix saposhnikoviae (6 g) • Radix gentianae macrophyllae (6 g) • Ramulus loranthi (6 g) • Radix angelicae pubescentis (9 g)	• Vitro: 300 μg/mL	• -	• -	• -	-	• Inhibit apoptosis	• CXCR4/NF-κB	Liu et al. (2018)

#### 2.2.3 Other TCM metabolites

In addition to the well-known Bushen Huoxue Formula and Duhuo Jisheng Decoction, recent studies have further expanded other TCM metabolites in the treatment of IVDD. In 2021, Dai et al. (Dai et al., 2021) demonstrated that Yiqi Huoxue Recipe could promote the formation of the Beclin1-VPS34 complex by activating the upstream protein AMP-activated protein kinase (AMPK) and up-regulating the deubiquitinase ubiquitin specific peptidase 13 (USP13). This activation of autophagy attenuated the release of inflammatory factors in NPCs. This study introduced the potential molecular mechanism of Yiqi Huoxue Formula’s autophagic action for the first time, providing both theoretical and experimental support for its clinical application in treating IVDD-related conditions. In 2025, another study confirmed the effective active metabolites and signaling pathways of Fuzi Decoction in alleviating IVDD through network pharmacological analysis. Subsequent cell and animal experiments validated these findings. The results revealed that Fuzi Decoction could regulate ECM degradation and ferroptosis by inhibiting the NF-κB signaling pathway, demonstrating its potential in mitigating IVDD. These findings suggest that Fuzi Decoction could be a promising therapeutic agent for IVDD, offering valuable insights into its multifaceted mechanisms and molecular interactions for future clinical applications (Liu Y. et al., 2025).

**FIGURE 7:**
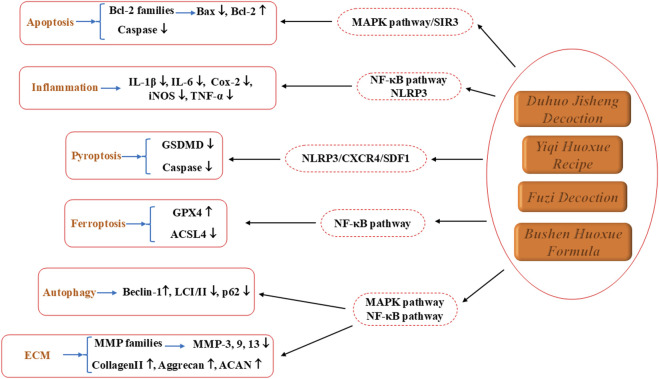
The therapeutic effects and underlying mechanism of TCM metabolite on IVDD.

The above researches specifically elaborate the pharmacological effects of various TCM metabolites in the context of IVDD, as illustrated in Figure 7. Looking ahead, further development and exploration of the pharmacological effects in IVDD are anticipated. By combining modern preparation technologies to improve bioavailability, along with minimally invasive interventions and rehabilitation physiotherapy, a comprehensive treatment approach integrating both traditional Chinese and Western medicine can be formed. This integrated model is expected to offer a safer and more effective treatment pathway for patients with IVDD.

## 3 Limitations

The intervertebral disc, being an avascular and enclosed tissue, makes it difficult to deliver the drug. The bioavailability of the active metabolite after systemic administration is extremely low, and it is difficult to penetrate the annulus fibrosus barrier to reach an effective concentration at the lesion site, resulting in an impractical dose *in vivo* even when the dose is effective *in vitro*. Secondly, Some of the metabolites studied may fall under the category of pan assay interfering compounds (PAINS). This means that the “activity” observed in in vitro experiments may result from non-specific interference mechanisms, including aggregation, redox reactions, fluorescence quenching rather than specific interactions with specific targets. Therefore, the reported *in vitro* activity results of such metabolites need to be interpreted with extreme caution, and their prospects as potential drug candidates are thus greatly diminished. Furthermore, most research results are based on cell or animal models, which cannot truly simulate the unique mechanical and physical microenvironment of human intervertebral discs. The studies generally lack direct comparisons with standard therapies and phased efficacy evaluations, making their clinical value unclear. Finally, the BMBDs are subject to variations due to the location, collection, and processing methods, resulting in inherent heterogeneity. This poses significant challenges for standardization, dosage determination, and the reproducibility of therapeutic effects.

In conclusion, it is necessary to clearly recognize its limitations in terms of delivery, efficacy, evidence, and standardization. In the future, efforts should be made to develop new delivery systems, explore combination therapies, and promote its clinical application through rigorous research.

## 4 Prospects

To promote the clinical application of BMBDs in the treatment of IVDD, future research needs to make breakthroughs in the following key areas. Firstly, developing advanced drug delivery systems is the core challenge. In the future, efforts should be focused on researching targeted delivery systems based on nanotechnology or hydrogels, in order to achieve precise, sustained-release and efficient enrichment of active metabolites at the lesion site, thereby addressing the fundamental problem of low bioavailability. Secondly, conduct in-depth research on the mechanism of multi-metabolites synergy. In the future, we should not limit ourselves to a single metabolite, but instead explore the scientific combination of multiple active metabolites. Utilize systems pharmacology and high-throughput screening techniques to construct a more potent multi-target regulatory network with higher efficacy. Finally, establish a more reliable paradigm for translational medical research. It is urgent to develop 3D culture or organ-on-a-chip models that can simulate the mechanical and biochemical microenvironment of human intervertebral discs, and promote their transformation into standardized drugs.

## 5 Conclusion

This review summarizes the great potential of various BMBDs in the treatment of intervertebral disc protrusion (IVDD), including a series of *in vivo* and *in vitro* experiments, multiple physiological processes, key signaling pathways and molecular mechanisms. As the therapeutic effects of BMBDs in treating IVDD have gained increasing recognition, it is expected to become a new treatment method, providing promising possibilities for the development of future drugs. However, its clinical application is still limited by some core challenges, such as low delivery efficiency, possibility of interference from PAINS, and difficulties in standardizing the metabolites. In the future, it is necessary to break through the targeted delivery technology, clarify the synergistic mechanism of multiple metabolites, and establish evidence-based medical evidence to realize its clinical translational value.

## References

[B1] Bai X. Jiang M. Wang J. Yang S. Liu Z. Zhang H. (2022a). Cyanidin attenuates the apoptosis of rat nucleus pulposus cells and the degeneration of intervertebral disc *via* the JAK2/STAT3 signal pathway *in vitro* and *in vivo* . Pharm. Biol. 60, 427–436. 10.1080/13880209.2022.2035773 35175176 PMC8856032

[B2] Bai X. Lian Y. Hu C. Yang S. Pei B. Yao M. (2022b). Cyanidin-3-glucoside protects against high glucose-induced injury in human nucleus pulposus cells by regulating the Nrf2/HO-1 signaling. J. Appl. Toxicol. 42, 1137–1145. 10.1002/jat.4281 34964128

[B3] Carrillo-Martinez E. J. Flores-Hernández F. Y. Salazar-Montes A. M. Nario-Chaidez H. F. Hernández-Ortega L. D. (2024). Quercetin, a flavonoid with great pharmacological capacity. Molecules 29 (5), 1000. 10.3390/molecules29051000 38474512 PMC10935205

[B4] Chen E. Li M. Liao Z. Yao D. Li Y. Huang L. (2024). Phillyrin reduces ROS production to alleviate the progression of intervertebral disc degeneration by inhibiting NF-κB pathway. J. Orthop. Surg. Res. 19 (1), 308. 10.1186/s13018-024-04695-y 38773639 PMC11110443

[B5] Chen S. Dou Y. Zhang Y. Sun X. Liu X. Yang Q. (2025). Innovating intervertebral disc degeneration therapy: harnessing the power of extracellular vesicles. J. Orthop. Transl. 50, 44–55. 10.1016/j.jot.2024.09.014 39868351 PMC11761297

[B6] Cherif H. Bisson D. G. Jarzem P. Weber M. Ouellet J. A. Haglund L. (2019). Curcumin and o-Vanillin exhibit evidence of senolytic activity in human IVD cells *in vitro* . J. Clin. Med. 8 (4), 433. 10.3390/jcm8040433 30934902 PMC6518239

[B7] Corrêa Carvalho G. Marena G. D. Gaspar Gonçalves Fernandes M. Ricci Leonardi G. Santos H. A. Chorilli M. (2024). Curcuma longa: nutraceutical use and association with nanotechnology. Adv. Healthc. Mater 13 (22), e2400506. 10.1002/adhm.202400506 38712468

[B8] Dai F. Yu P. Yu Z. Jiang H. Ma Z. Liu J. (2021). Yiqi huoxue recipe delayed intervertebral disc degeneration by activating autophagy. Front. Pharmacol. 12, 705747. 10.3389/fphar.2021.705747 34483910 PMC8416448

[B9] Deng X. Chen S. Zheng D. Shao Z. Liang H. Hu H. (2017). Icariin prevents H(2)O(2)-Induced apoptosis *via* the PI3K/Akt pathway in Rat Nucleus Pulposus Intervertebral disc cells. Evid. Based Complement. Altern. Med. 2017, 2694261. 10.1155/2017/2694261 28536643 PMC5425849

[B10] Dou Y. Zhang Y. Liu Y. Sun X. Liu X. Li B. (2025). Role of macrophage in intervertebral disc degeneration. Bone Res. 13, 15. 10.1038/s41413-024-00397-7 39848963 PMC11758090

[B11] Duan J. Li Z. Liu E. Long H. Chen L. Yang S. (2023). BSHXF-medicated serum combined with ADSCs regulates the TGF-β1/Smad pathway to repair oxidatively damaged NPCs and its component analysis. J. Ethnopharmacol. 316, 116692. 10.1016/j.jep.2023.116692 37277086

[B12] Fang W. Zhou X. Wang J. Xu L. Zhou L. Yu W. (2018). Wogonin mitigates intervertebral disc degeneration through the Nrf2/ARE and MAPK signaling pathways. Int. Immunopharmacol. 65, 539–549. 10.1016/j.intimp.2018.10.024 30412851

[B13] Gan Y. He J. Gong Y. Wu Z. Liang D. Shen G. (2025). Baicalein-loaded porous silk fibroin microspheres modulate the senescence of nucleus pulposus cells through the NF-κB signaling pathway. Colloids Surf. B Biointerfaces 249, 114537. 10.1016/j.colsurfb.2025.114537 39879672

[B14] Gao Z. Lin Y. Zhang P. Cheng Q. Ye L. Wu F. (2019). Sinomenine ameliorates intervertebral disc degeneration *via* inhibition of apoptosis and autophagy *in vitro* and *in vivo* . Am. J. Transl. Res. 11, 5956–5966. 31632563 PMC6789258

[B15] Gao S. Li N. Chen R. Su Y. Song Y. Liang S. (2022). Bushen huoxue formula modulates Autophagic Flux and inhibits apoptosis to protect Nucleus Pulposus cells by restoring the AMPK/SIRT1 pathway. Biomed. Res. Int. 2022, 8929448. 10.1155/2022/8929448 35669720 PMC9167005

[B16] Gao S. Wang C. Qi L. Liang S. Qu X. Liu W. (2024). Bushen huoxue formula inhibits IL-1β-Induced apoptosis and extracellular matrix degradation in the Nucleus Pulposus cells and improves intervertebral disc degeneration in rats. J. Inflamm. Res. 17, 121–136. 10.2147/JIR.S431609 38204990 PMC10777862

[B17] Guo D. Cheng K. Song C. Liu F. Cai W. Chen J. (2023). Mechanisms of inhibition of nucleus pulposus cells pyroptosis through SDF1/CXCR4-NFkB-NLRP3 axis in the treatment of intervertebral disc degeneration by Duhuo Jisheng Decoction. Int. Immunopharmacol. 124 (Pt A), 110844. 10.1016/j.intimp.2023.110844 37647678

[B18] He S. Fu Y. Yan B. Tan H. Li H. Li J. (2021). Curcumol alleviates the inflammation of Nucleus Pulposus cells *via* the PI3K/Akt/NF-κB signaling pathway and delays intervertebral disk degeneration. World Neurosurg. 155, e402–e411. 10.1016/j.wneu.2021.08.079 34450323

[B19] Hu Y. Tang J. S. Hou S. X. Shi X. X. Qin J. Zhang T. S. (2017). Neuroprotective effects of curcumin alleviate lumbar intervertebral disc degeneration through regulating the expression of iNOS, COX-2, TGF-β1/2, MMP-9 and BDNF in a rat model. Mol. Med. Rep. 16 (5), 6864–6869. 10.3892/mmr.2017.7464 28901458

[B20] Hu B. Lin S. Lin S. Rui G. (2023). Ginkgetin alleviates intervertebral disc degeneration by inhibiting apoptosis, inflammation, and disturbance of extracellular matrix synthesis and catabolism *via* inactivation of NLRP3 inflammasome. Immunol. Invest. 52, 546–560. 10.1080/08820139.2023.2205884 37154418

[B21] Hua W. Zhang Y. Wu X. Kang L. Tu J. Zhao K. (2018). Icariin Attenuates Interleukin-1β-Induced inflammatory response in Human Nucleus Pulposus cells. Curr. Pharm. Des. 23, 6071–6078. 10.2174/1381612823666170615112158 28619001

[B22] Huang L. Chen J. Wu D. Wang K. Lou W. Wu J. (2022). Berberine Attenuates IL-1β-Induced damage of Nucleus Pulposus cells *via* activating the AMPK/mTOR/Ulk1 pathway. Biomed. Res. Int. 2022, 6133629. 10.1155/2022/6133629 35915801 PMC9338861

[B23] Ji Z. Guo R. Ma Z. Li H. (2022). Arctigenin inhibits apoptosis, extracellular matrix degradation, and inflammation in human nucleus pulposus cells by up-regulating miR-483-3p. J. Clin. Lab. Anal. 36, e24508. 10.1002/jcla.24508 35689566 PMC9280009

[B24] Jin H. Wang Q. Wu J. Han X. Qian T. Zhang Z. (2019). Baicalein inhibits the IL-1β-Induced inflammatory response in Nucleus Pulposus cells and attenuates disc degeneration *in vivo* . Inflammation 42 (3), 1032–1044. 10.1007/s10753-019-00965-8 30729381

[B25] Kamal R. Paul P. Thakur S. Singh S. K. Awasthi A. (2024). Quercetin in oncology: a phytochemical with immense therapeutic potential. Curr. Drug Targets 25 (11), 740–751. 10.2174/0113894501292466240627050638 38988154

[B26] Kim J. H. Ham C. H. Kwon W. K. (2022). Current knowledge and future therapeutic prospects in symptomatic intervertebral disc degeneration. Yonsei Med. J. 63 (3), 199–210. 10.3349/ymj.2022.63.3.199 35184422 PMC8860939

[B27] Klawitter M. Quero L. Klasen J. Gloess A. N. Klopprogge B. Hausmann O. (2012). Curcuma DMSO extracts and curcumin exhibit an anti-inflammatory and anti-catabolic effect on human intervertebral disc cells, possibly by influencing TLR2 expression and JNK activity. J. Inflamm. (Lond). 9 (1), 29. 10.1186/1476-9255-9-29 22909087 PMC3506446

[B28] Kotha R. R. Luthria D. L. (2019). Curcumin: biological, pharmaceutical, nutraceutical, and analytical aspects. Molecules 24 (16), 2930. 10.3390/molecules24162930 31412624 PMC6720683

[B29] Li W. Zhang Y. Xing C. Zhang M. (2015). Tanshinone IIA represses inflammatory response and reduces radiculopathic pain by inhibiting IRAK-1 and NF-κB/p38/JNK signaling. Int. Immunopharmacol. 28, 382–389. 10.1016/j.intimp.2015.06.032 26163178

[B30] Li N. Whitaker C. Xu Z. Heggeness M. Yang S. Y. (2016). Therapeutic effects of naringin on degenerative human nucleus pulposus cells for discogenic low back pain. Spine J. 16, 1231–1237. 10.1016/j.spinee.2016.05.007 27208552

[B31] Li Z. Wang Y. Xu Q. Ma J. Li X. Yan J. (2023). Berberine and health outcomes: an umbrella review. Phytother. Res. 37 (5), 2051–2066. 10.1002/ptr.7806 36999891

[B32] Li M. Yu X. Chen X. Jiang Y. Zeng Y. Ren R. (2024). Genkwanin alleviates intervertebral disc degeneration *via* regulating ITGA2/PI3K/AKT pathway and inhibiting apoptosis and senescence. Int. Immunopharmacol. 133, 112101. 10.1016/j.intimp.2024.112101 38640717

[B33] Li Z. Cheng P. Xi H. Jiang T. Zheng X. Qiu J. (2024). Tomatidine alleviates intervertebral disc degeneration by activating the Nrf2/HO-1/GPX4 signaling pathway. Drug Des. Devel Ther. 18, 6313–6329. 10.2147/DDDT.S481714 39741916 PMC11687091

[B34] Liu Z. C. Jiang Y. Huang C. Y. Liu Y. Wei Z. C. Liu S. G. (2018). Mechanism of Duhuo Jisheng decotion in delaying degeneration of nucleus pulposus cells in human intervertebral disc. Zhongguo Zhong Yao Za Zhi 43 (13), 2764–2769. 10.19540/j.cnki.cjcmm.20180503.001 30111029

[B35] Liu Z. C. Wang Z. L. Huang C. Y. Fu Z. J. Liu Y. Wei Z. C. (2018). Duhuo Jisheng Decoction inhibits SDF-1-induced inflammation and matrix degradation in human degenerative nucleus pulposus cells *in vitro* through the CXCR4/NF-κB pathway. Acta Pharmacol. Sin. 39 (6), 912–922. 10.1038/aps.2018.36 29795361 PMC6256264

[B36] Liu W. Jin S. Huang M. Li Y. Wang Z. Wang P. (2020). Duhuo jisheng decoction suppresses matrix degradation and apoptosis in human nucleus pulposus cells and ameliorates disc degeneration in a rat model. J. Ethnopharmacol. 250, 112494. 10.1016/j.jep.2019.112494 31874213

[B37] Liu Y. Zheng J. Chen Y. Wang F. Ye H. Wang M. (2021). Shikonin protects against lipopolysaccharide-induced inflammation and apoptosis in human nucleus pulposus cells through the nuclear factor-kappa B pathway. Food Sci. Nutr. 9 (10), 5583–5589. 10.1002/fsn3.2519 34646528 PMC8497831

[B38] Liu W. Zhao X. Wu X. (2023). Duhuo Jisheng Decoction suppresses apoptosis and mitochondrial dysfunction in human nucleus pulposus cells by miR-494/SIRT3/mitophagy signal axis. J. Orthop. Surg. Res. 18 (1), 177. 10.1186/s13018-023-03669-w 36890588 PMC9996943

[B39] Liu Y. Liu D. K. Wang Z. W. Zhao C. Miao J. (2023). Baicalein alleviates TNF-α-induced apoptosis of human nucleus pulposus cells through PI3K/AKT signaling pathway. J. Orthop. Surg. Res. 18 (1), 292. 10.1186/s13018-023-03759-9 37041597 PMC10088118

[B40] Liu R. Xu L. X. Tong L. J. Wu H. Y. Guo Q. Sun Z. M. (2024). Therapeutic effects of ginsenosides on osteoporosis for novel drug applications. Eur. J. Pharmacol. 974, 176604. 10.1016/j.ejphar.2024.176604 38649090

[B41] Liu X. Pan F. Sha C. Wang Z. Liu G. Wang H. (2025). Fuzi decoction ameliorates intervertebral disc degeneration through ferroptosis modulation by suppressing NF-κB pathway. Int. Immunopharmacol. 148, 114155. 10.1016/j.intimp.2025.114155 39874850

[B42] Liu Y. Huang T. Wang L. Wang Y. Bai J. (2025). Traditional Chinese Medicine in the treatment of chronic atrophic gastritis, precancerous lesions and gastric cancer. J. Ethnopharmacol. 337 (Pt 1), 118812. 10.1016/j.jep.2024.118812 39260710

[B43] Lou J. Ryan R. Wang D. (2025). Biologic therapies for discogenic pain. Curr. Pain Headache Rep. 29 (1), 45. 10.1007/s11916-024-01325-4 39932512

[B44] Lu L. Hu J. Wu Q. An Y. Cui W. Wang J. (2019). Berberine prevents human nucleus pulposus cells from IL-1β-induced extracellular matrix degradation and apoptosis by inhibiting the NF-κB pathway. Int. J. Mol. Med. 43 (4), 1679–1686. 10.3892/ijmm.2019.4105 30816449 PMC6414164

[B45] Luo R. Liao Z. Song Y. Yin H. Zhan S. Li G. (2019). Berberine ameliorates oxidative stress-induced apoptosis by modulating ER stress and autophagy in human nucleus pulposus cells. Life Sci. 228, 85–97. 10.1016/j.lfs.2019.04.064 31047897

[B46] Mai Y. Wu S. Zhang P. Chen N. Wu J. Wei F. (2025). The anti-oxidation related bioactive materials for intervertebral disc degeneration regeneration and repair. Bioact. Mater 45, 19–40. 10.1016/j.bioactmat.2024.10.012 39588482 PMC11585838

[B47] Mohd Isa I. L. Teoh S. L. Mohd Nor N. H. Mokhtar S. A. (2022). Discogenic low back pain: anatomy, pathophysiology and treatments of intervertebral disc degeneration. Int. J. Mol. Sci. 24 (1), 208. 10.3390/ijms24010208 36613651 PMC9820240

[B48] Qi L. Zhang J. Wang J. An J. Xue W. Liu Q. (2022). Mechanisms of ginsenosides exert neuroprotective effects on spinal cord injury: a promising traditional Chinese medicine. Front. Neurosci. 16, 969056. 10.3389/fnins.2022.969056 36081662 PMC9445311

[B49] Ren J. Xin R. Cui X. Xu Y. Li C. (2024). Quercetin relieves compression-induced cell death and lumbar disc degeneration by stabilizing HIF1A protein. Heliyon 10 (17), e37349. 10.1016/j.heliyon.2024.e37349 39296087 PMC11408125

[B50] Shao Z. Wang B. Shi Y. Xie C. Huang C. Chen B. (2021). Senolytic agent Quercetin ameliorates intervertebral disc degeneration *via* the Nrf2/NF-κB axis. Osteoarthr. Cartil. 29 (3), 413–422. 10.1016/j.joca.2020.11.006 33242601

[B51] Shao T. Gao Q. Ma Y. Gu J. Yu Z. (2024). Hyperforin improves matrix stiffness induced nucleus pulposus inflammatory degeneration by activating mitochondrial fission. Int. Immunopharmacol. 137, 112444. 10.1016/j.intimp.2024.112444 38901245

[B52] Sono T. Shima K. Shimizu T. Murata K. Matsuda S. Otsuki B. (2024). Regenerative therapies for lumbar degenerative disc diseases: a literature review. Front. Bioeng. Biotechnol. 12, 1417600. 10.3389/fbioe.2024.1417600 39257444 PMC11385613

[B53] Su X. Liu B. Gong F. Yin J. Sun Q. Gao Y. (2019). Isofraxidin attenuates IL-1β-induced inflammatory response in human nucleus pulposus cells. J. Cell Biochem. 120 (8), 13302–13309. 10.1002/jcb.28604 30891836

[B54] Tang K. Su W. Huang C. Wu Y. Wu X. Lu H. (2021). Notoginsenoside R1 suppresses inflammatory response and the pyroptosis of nucleus pulposus cells *via* inactivating NF-κB/NLRP3 pathways. Int. Immunopharmacol. 101 (Pt B), 107866. 10.1016/j.intimp.2021.107866 34588155

[B55] Tang H. Zhang S. Lu X. Geng T. (2023). Effects of puerarin on the intervertebral disc degeneration and biological characteristics of nucleus pulposus cells. Pharm. Biol. 61 (1), 12–22. 10.1080/13880209.2022.2147548 36524765 PMC9762855

[B56] Tang X. Zhong J. Luo H. Zhou F. Wang L. Lin S. (2025). Efficacy of Naringenin against aging and degeneration of nucleus pulposus cells through IGFBP3 inhibition. Sci. Rep. 15, 6780. 10.1038/s41598-025-90909-0 40000729 PMC11861589

[B57] Tian Y. Chu X. Huang Q. Guo X. Xue Y. Deng W. (2022). Astragaloside IV attenuates IL-1β-induced intervertebral disc degeneration through inhibition of the NF-κB pathway. J. Orthop. Surg. Res. 17 (1), 545. 10.1186/s13018-022-03438-1 36527065 PMC9758796

[B58] Tsuchiya K. Okano I. Guven A. E. Verna B. Köhli P. Hambrecht J. (2025). Quantitative assessment of cervical disc degeneration using disc signal intensity index. Spine J. 25, 903–910. 10.1016/j.spinee.2024.11.017 39645168

[B59] Wan C. Liu S. Zhao L. Chang C. Li H. Li R. (2025). Curcumin protects rat endplate chondrocytes against IL-1β-induced apoptosis *via* Bcl-2/Bax regulation. J. Mol. Histol. 56 (2), 111. 10.1007/s10735-025-10390-x 40106034

[B60] Wang J. Huang C. Lin Z. Pan X. Chen J. Zheng G. (2018). Polydatin suppresses nucleus pulposus cell senescence, promotes matrix homeostasis and attenuates intervertebral disc degeneration in rats. J. Cell Mol. Med. 22, 5720–5731. 10.1111/jcmm.13848 30358118 PMC6201341

[B61] Wang K. Chen T. Ying X. Zhang Z. Shao Z. Lin J. (2019). Ligustilide alleviated IL-1β induced apoptosis and extracellular matrix degradation of nucleus pulposus cells and attenuates intervertebral disc degeneration *in vivo* . Int. Immunopharmacol. 69, 398–407. 10.1016/j.intimp.2019.01.004 30785069

[B62] Wang D. He X. Wang D. Peng P. Xu X. Gao B. (2020). Quercetin suppresses apoptosis and attenuates intervertebral disc degeneration *via* the SIRT1-Autophagy pathway. Front. Cell Dev. Biol. 8, 613006. 10.3389/fcell.2020.613006 33363176 PMC7758489

[B63] Wang D. Qu H. Kang H. Xu F. Huang W. Cai X. (2022). Kukoamine A attenuates lipopolysaccharide-induced apoptosis, extracellular matrix degradation, and inflammation in nucleus pulposus cells by activating the P13K/Akt pathway. Bioengineered 13 (4), 8772–8784. 10.1080/21655979.2022.2051855 35333664 PMC9161835

[B64] Wang J. Zou J. Shi Y. Zeng N. Guo D. Wang H. (2024). Traditional Chinese medicine and mitophagy: a novel approach for cardiovascular disease management. Phytomedicine 128, 155472. 10.1016/j.phymed.2024.155472 38461630

[B65] Wang X. Huang Y. Yang Y. Tian X. Jin Y. Jiang W. (2025). Polysaccharide-based biomaterials for regenerative therapy in intervertebral disc degeneration. Mater Today Bio 30, 101395. 10.1016/j.mtbio.2024.101395 39759846 PMC11699348

[B66] Wen R. J. Dong X. Zhuang H. W. Pang F. X. Ding S. C. Li N. (2023). Baicalin induces ferroptosis in osteosarcomas through a novel Nrf2/xCT/GPX4 regulatory axis. Phytomedicine 116, 154881. 10.1016/j.phymed.2023.154881 37209607

[B67] Wu X. Pan Q. Yao C. Gong Y. Li Z. Tang F. (2025). Therapeutic potential of quercitrin in intervertebral disc degeneration: targeting pyroptosis and inflammation. Int. Immunopharmacol. 156, 114680. 10.1016/j.intimp.2025.114680 40273673

[B68] Wu Y. Li F. Shu S. Feng Z. Qiu Y. Li S. (2025). Baicalin alleviates intervertebral disc degeneration by inhibiting the p38 MAPK signaling pathway. Exp. Gerontol. 204, 112743. 10.1016/j.exger.2025.112743 40174870

[B69] Wu Z. L. Liu Y. Song W. Zhou K. S. Ling Y. Zhang H. H. (2025). Role of mitophagy in intervertebral disc degeneration: a narrative review. Osteoarthr. Cartil. 33 (1), 27–41. 10.1016/j.joca.2024.09.013 39537018

[B70] Xie T. Yuan J. Mei L. Li P. Pan R. (2022). Hyperoside ameliorates TNF-α-induced inflammation, ECM degradation and ER stress-mediated apoptosis *via* the SIRT1/NF-κB and Nrf2/ARE signaling pathways *in vitro* . Mol. Med. Rep. 26 (2), 260. 10.3892/mmr.2022.12776 35730622 PMC9260875

[B71] Xu Y. He J. He J. (2022). Cyanidin attenuates the high hydrostatic pressure-induced degradation of cellular matrix of nucleus pulposus cell *via* blocking the Wnt/β-catenin signaling. Tissue Cell 76, 101798. 10.1016/j.tice.2022.101798 35472676

[B72] Yang S. Li L. Zhu L. Zhang C. Li Z. Guo Y. (2019). Bu-Shen-Huo-Xue-Fang modulates nucleus pulposus cell proliferation and extracellular matrix remodeling in intervertebral disk degeneration through miR-483 regulation of Wnt pathway. J. Cell Biochem. 120 (12), 19318–19329. 10.1002/jcb.26760 29393545

[B73] Yang Y. H. Gu X. P. Hu H. Hu B. Wan X. L. Gu Z. P. (2022). Ginsenoside Rg1 inhibits nucleus pulposus cell apoptosis, inflammation and extracellular matrix degradation *via* the YAP1/TAZ pathway in rats with intervertebral disc degeneration. J. Orthop. Surg. Res. 17 (1), 555. 10.1186/s13018-022-03443-4 36539815 PMC9768949

[B74] Yang S. Zhu Y. Shi Y. Su S. Liang H. Li S. (2024). Screening of NSAIDs library identifies Tinoridine as a novel ferroptosis inhibitor for potential intervertebral disc degeneration therapy. Free Radic. Biol. Med. 221, 245–256. 10.1016/j.freeradbiomed.2024.05.040 38806104

[B75] Yao D. Li M. Zeng W. Wang K. Liao Z. Chen E. (2025). LRP1 mitigates intervertebral disc degeneration by inhibiting endoplasmic reticulum stress through stabilizing the PPARγ. J. Orthop. Transl. 50, 196–210. 10.1016/j.jot.2024.12.009 39895867 PMC11786795

[B76] Yu L. Hao Y. Peng C. Zhang P. Zhu J. Cai Y. (2020). Effect of Ginsenoside Rg1 on the intervertebral disc degeneration rats and the degenerative pulposus cells and its mechanism. Biomed. Pharmacother. 123, 109738. 10.1016/j.biopha.2019.109738 31951975

[B77] Yu L. Hao Y. J. Ren Z. N. Zhu G. D. Zhou W. W. Lian X. (2024). Ginsenoside Rg1 relieves rat intervertebral disc degeneration and inhibits IL-1β-induced nucleus pulposus cell apoptosis and inflammation *via* NF-κB signaling pathway. Vitro Cell Dev. Biol. Anim. 60 (3), 287–299. 10.1007/s11626-024-00883-6 38485818 PMC11014818

[B78] Yu H. Chen K. Li X. Liang J. Jin Y. Bao Y. (2025). Palmatine activation of TFEB enhances autophagy and alleviates endoplasmic reticulum stress in intervertebral disc degeneration. Phytomedicine 139, 156431. 10.1016/j.phymed.2025.156431 39933468

[B79] Yu Z. Wang X. Hu Y. Zhu Z. Xu X. (2025). Quercetin alleviates hyperglycemic-generated endoplasmic reticulum stress-contacted apoptosis of Rat Nucleus Pulposus cells. Balk. Med. J. 42 (1), 27–36. 10.4274/balkanmedj.galenos.2024.2024-7-92 39757454 PMC11725678

[B80] Zàaba N. F. Ogaili R. H. Ahmad F. Mohd Isa I. L. (2025). Neuroinflammation and nociception in intervertebral disc degeneration: a review of precision medicine perspective. Spine J. 25, 1139–1153. 10.1016/j.spinee.2024.12.033 39814205

[B81] Zhan J. W. Li K. M. Zhu L. G. Wang S. Q. Feng M. S. Wei X. (2022). Efficacy and safety of Bushen huoxue formula in patients with discogenic low-back pain: a Double-Blind, randomized, placebo-controlled trial. Chin. J. Integr. Med. 28 (11), 963–970. 10.1007/s11655-022-3505-4 35840851

[B82] Zhan J. Cui Y. Zhang P. Du Y. Hecker P. Zhou S. (2025). Cartilage endplate-targeted engineered exosome releasing and acid neutralizing Hydrogel reverses intervertebral disc degeneration. Adv. Healthc. Mater 14, e2403315. 10.1002/adhm.202403315 39555665

[B83] Zhang L. Chen Q. Wang H. Yang J. Sheng S. (2018). Andrographolide mitigates IL-1β-induced human nucleus pulposus cells degeneration through the TLR4/MyD88/NF-κB signaling pathway. Mol. Med. Rep. 18 (6), 5427–5436. 10.3892/mmr.2018.9599 30365119 PMC6236278

[B84] Zhang W. J. Liu Y. Wei J. S. Wu Y. L. (2019). Ginsenoside Rd inhibits IL-1β-induced inflammation and degradation of intervertebral disc chondrocytes by increasing IL1RAP ubiquitination. Braz J. Med. Biol. Res. 52 (9), e8525. 10.1590/1414-431X20198525 31411316 PMC6694592

[B85] Zhang S. Liang W. Abulizi Y. Xu T. Cao R. Xun C. (2021). Quercetin alleviates intervertebral disc degeneration by modulating p38 MAPK-Mediated autophagy. Biomed. Res. Int. 2021, 6631562. 10.1155/2021/6631562 34055990 PMC8133869

[B86] Zhao K. Chen M. Liu T. Zhang P. Wang S. Liu X. (2021). Rhizoma drynariae total flavonoids inhibit the inflammatory response and matrix degeneration *via* MAPK pathway in a rat degenerative cervical intervertebral disc model. Biomed. Pharmacother. 138, 111466. 10.1016/j.biopha.2021.111466 33740525

[B87] Zhao W. J. Liu X. Hu M. Zhang Y. Shi P. Z. Wang J. W. (2023). Quercetin ameliorates oxidative stress-induced senescence in rat nucleus pulposus-derived mesenchymal stem cells *via* the miR-34a-5p/SIRT1 axis. World J. Stem Cells 15 (8), 842–865. 10.4252/wjsc.v15.i8.842 37700818 PMC10494568

[B88] Zhou Y. Chen Z. Yang X. Cao X. Liang Z. Ma H. (2021). Morin attenuates pyroptosis of nucleus pulposus cells and ameliorates intervertebral disc degeneration *via* inhibition of the TXNIP/NLRP3/Caspase-1/IL-1β signaling pathway. Biochem. Biophys. Res. Commun. 559, 106–112. 10.1016/j.bbrc.2021.04.090 33933989

[B89] Zhou D. Song C. Mei Y. Cheng K. Liu F. Cai W. (2023). A review of Duhuo Jisheng decoction mechanisms in intervertebral disc degeneration *in vitro* and animal studies. J. Orthop. Surg. Res. 18 (1), 436. 10.1186/s13018-023-03869-4 37322524 PMC10273736

[B90] Zhu X. Guo S. Zhang M. Bai X. (2023). Emodin protects against apoptosis and inflammation by regulating reactive oxygen species-mediated NF-κB signaling in interleukin-1β-stimulated human nucleus pulposus cells. Hum. Exp. Toxicol. 42, 9603271221138552. 10.1177/09603271221138552 36598795

